# Thyroid ultrasound pattern in primary hypothyroidism is similar to Graves’ disease: a report of three cases

**DOI:** 10.25122/jml-2023-0507

**Published:** 2024-01

**Authors:** Andrey Valerievich Ushakov

**Affiliations:** 1 Ushakov Thyroid Clinic, Moscow, Russia

**Keywords:** thyroid ultrasound, hypothyroidism, hyperthyroidism, Graves’ disease, autonomic nervous system, TSHR-Ab, Doppler, PSV-STA, FT3, free triiodothyronine, FT4, free thyroxine, GD, Graves’ disease, PSV, peak systolic velocity, STA, superior thyroid arteries, TGAb, thyroglobulin, Total T4, total thyroxine, TPOAb, thyroid peroxidase, TSH, thyroid-stimulating hormone

## Abstract

Ultrasound can identify important characteristics in primary hypothyroidism and diffuse hyperthyroidism (Graves’ disease). Therefore, sonologists are actively investigating ultrasound criteria to differentiate between these two conditions. Nevertheless, practice shows the absence of such ultrasonic landmarks. For the first time in the literature, three cases of primary hypothyroidism have demonstrated an ultrasound pattern identical to that of Graves’ disease. This pattern includes the presence of goiter, marked total hypoechogenicity of the parenchyma, significantly or moderately increased blood flow intensity (‘thyroid inferno’), and elevated peak systolic velocity of the superior thyroid arteries. These signs are less common in hypothyroidism compared to hyperthyroidism. Diagnostic data suggest that the pathogeneses of primary hypothyroidism and Graves’ disease share the same mechanisms, leading to similar thyroid ultrasound patterns. One of these shared mechanisms is presumably thyroid overstimulation by the autonomic nervous system, which is adequate to the body’s hormonal requirements in hypothyroidism but excessive in hyperthyroidism.

## INTRODUCTION

Before the advent of medical ultrasound, goiter was known to occur in hypothyroidism, euthyroidism, and hyperthyroidism. However, ultrasound demonstrated three additional diagnostic features of the thyroid in these hormonal conditions: hypoechogenicity of the parenchyma, and increased blood flow intensity and velocity in the inflow arteries.

Almost 2 years after the introduction of color Doppler imaging, an article by Ralls *et al*. on the ultrasound features of Graves’ disease (GD) mentioned “a pulsatile pattern”, which the authors called “thyroid inferno” [1]. There were no individuals with primary hypothyroidism among the examined patients in that study, and the patient groups were small. Nevertheless, the term ‘thyroid inferno’, used as a sign for hyperthyroidism, gained traction among specialists. Moreover, the unproven idea that intense blood flow in the thyroid corresponds only to GD was authoritatively accepted for many years [2,3].

Five years after the term ‘thyroid inferno’ was suggested as a sign of GD, Lagalla *et al*. identified ‘thyroid inferno’ in patients with hypothyroidism [4]. Their assumption regarding the hypertrophic role of thyroid-stimulating hormone (TSH) in thyroid hypervascularization remained unsubstantiated.

Studies on the differential diagnosis of GD using Doppler ultrasound have been performed mainly with destructive thyroiditis (Riedel’s disease), toxic multinodular goiter (Plummer’s disease), and Hashimoto’s thyroiditis [5,6]. However, most researchers have not identified and comprehensively characterized patients with primary hypothyroidism [5–7]. The authors likely assumed that Hashimoto’s thyroiditis is hypothyroidism, although the same diagnostic criteria of Hashimoto’s thyroiditis are also applied in GD, namely hypoechogenicity of the thyroid parenchyma and abundance of antibodies to thyroid peroxidase (TPOAb) and thyroglobulin (TGAb) [8]. Moreover, the histopathological features of Hashimoto’s thyroiditis are associated with diffuse heterogeneity on ultrasound and elevated antibodies only in 50–60% cases [9]. In addition, the authors statistically averaged the clinical and ultrasound signs of the thyroid in patients with hypothyroidism. Consequently, their conclusions revealed the presence of distinct Doppler criteria for distinguishing GD [5,7].

Nevertheless, some experts who were able to differentiate between patients with hypothyroidism observed increased thyroid vascularization and blood flow velocity in both GD and primary hypothyroidism [4,10–12]. Despite these similarities between different hormonal conditions, the conclusions about the mechanism of intensified thyroid blood flow in hypothyroidism were confined to assumptions about the role of TSH or TSH receptor antibodies (TRAb) [4,10]. However, this pathogenetic hypothesis has not been confirmed so far.

There is also a limitation in determining the cut-off value for peak systolic velocity (PSV) of the superior thyroid arteries (STA) in cases of hypothyroidism and GD. It is recognized that in primary hypothyroidism, PSV-STA can be almost 100% above the normal level, but they fail to explain the mechanism behind such an elevation [13,14].

The aim of this study was to identify the pathogenetic basis of thyroid changes common to hypothyroidism and hyperthyroidism. To our knowledge, this is the first study to describe cases of primary hypothyroidism with significantly increased blood flow intensity and PSV-STA, along with other ultrasound signs consistent with GD. We also describe for the first time the compensatory neurogenic nature of the pathogenesis of increased blood flow.

## CASE PRESENTATION

In March 2023, two patients with primary hypothyroidism presented to our clinic on the same day for diagnosis and consultation, and their ultrasound signs appeared similar to those commonly seen in GD. Although such cases do occur sometimes in our clinical practice, the remarkable coincidence of two cases presenting on the same day prompted us to highlight this variant of the disease. In January 2023, we had another similar case with ultrasonographic signs in manifested hypothyroidism. Therefore, we present all three cases together. The examination data of the patients are summarized in Table 1, with the medical history and ultrasound data provided for each case.

### Medical history of patient 1

In March 2022, the patient had COVID-19 with a fever of 39 ºC (axillary) for 5 days, anosmia for 7 days, and back muscle tension. A course of general sedative massage significantly improved her condition. In May 2022, a routine examination revealed euthyroidism and slightly raised TPOAb levels.

On 5 May 2022, TSH levels were 2.3 mIU/l (normal range, 0.34–5.60 mIU/l) and TPOAb levels were 153.9 (normal values, <9). On 10 May 2022, the following surgery under general anesthesia was performed: excision of excess fatty tissue and abdominal skin using mesh reinforcement. Over the next 7 days, the patient remained febrile and experienced significant weakness. Blood tests revealed a hemoglobin level of 60 g/dl (preoperatively 140 g/dl). The patient received blood transfusion for 2 days, with gradual normalization of the erythron. The course of ozone therapy significantly improved the patient’s condition.

**Figure 1:**
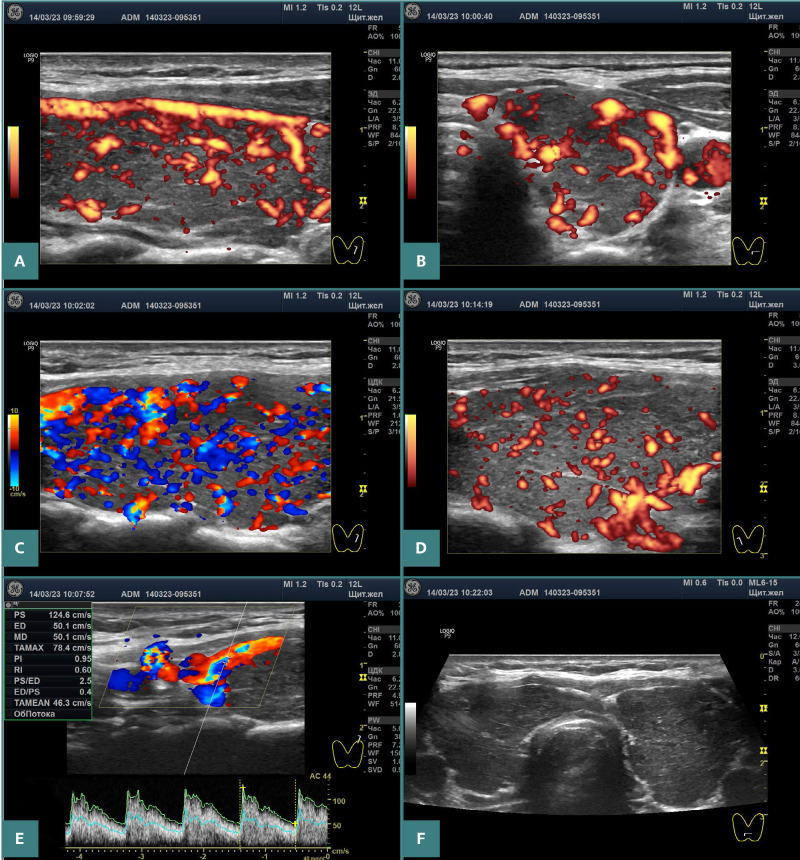
Ultrasound images of the thyroid of patient 1. A,B, Significant increase in blood flow intensity in the left lobe in power Doppler mode at optimal PRF. C, ‘Thyroid inferno’ sign in the left lobe in color Doppler mode at low PRF. D, Significant increase in blood flow intensity in the right lobe in power Doppler mode at optimal PRF. E, PSV in the first branch of the STAs on the left: 124.6 cm/s. F, Both lobes in grayscale mode.

However, on 28 October 2022, TSH levels were 1.2 mIU/l (normal range, 0.35–4.94) and TPOAb levels were 499 IU/l (normal values, <5.6). In February 2023, dragging pain in the lower abdomen appeared. Blood tests performed on 7 February 2023 revealed TSH levels of 1.3 mIU/l (normal range, 0.35–4.94) and TPOAb levels of 1,105.6 IU/l (normal values, <9). In February 2023, gastroduodenoscopy and colonoscopy were performed under anesthesia, which revealed no pathology. After this examination, the patient experienced a strong sensation of coldness in the chest and neck. The patient suspected that their thyroid might be affecting their wellbeing and presented to our clinic, where we discovered manifested hypothyroidism (Table 1), ultrasonographic signs of increased thyroid blood flow intensity and velocity (Figure 1A–E) and total hypoechogenicity of the parenchyma (Figure 1F).

**Table 1:** Examination data of the three patients

Variable	Patient 1 7 March 2023	Patient 2 11 March 2023	Patient 3 18 January 2023
Age, years	43	54	44
Height, cm	160	168	170
Body weight, kg	60	61	89
TSH, mIU/ml	33.8 (0.35–4.94)	10.3 (0.4–4.0)	22.0 (0.4–4.0)
FT4^a^	0.6 (0.70–1.48)	8.6 (9.00–19.05)	7.5 (9.00–19.05)
FT3, pmol/l	2.2 (1.58–3.91)	3.1 (3.0–5.6)	3.4 (3.0–5.6)
Total T4, nmol/l	49.1 (71–142)	66.1 (62.6–150.8)	52.8 (62.6–150.8)
TPOAb, IU/ml	1,654 (<34)	>1,000 (<5.6)	>1,000 (<5.6)
TGAb, IU/ml	60.3 (<4.11)	568 (<4.1)	52 (<4.1)
RBC, mln/mm^3^	4.7 (4.2–5.9)	4.6 (3.8–5.3)	4.2 (3.8–5.3)
Hemoglobin, g/dl	13.6 (13.0–18.0)	13.9 (11.7–16.0)	13.9 (11.7–16.0)
Complaints	Signs of gastric and intestinal dysregulation; generalized weakness throughout the day	Fatigue in the evening; sleep disturbance (wakes up at night for 1 h- three times a week); heart palpitations when climbing to the second floor	Fatigue
Thyroid volume, ml	26.5 (14.6 + 11.9)	39.0 (20.2 + 18.8)	18.1 (7.7 + 10.4)
Echogenicity of two lobes of parenchyma	Markedly hypoechogenic	Markedly hypoechogenic	Markedly hypoechogenic
PSV in the system of the superior thyroid artery, cm/s (right; left)	–; 124.6	96.5; 95.4	119.0; 160.1
PSV of the common carotid artery, cm/s (right; left)	50.1; 51.3	46.8; 42.3	61.1; 60.1
Blood flow intensity in parenchyma of thyroid lobes	Significantly increased	Moderately increased	Moderately increased
Use of thyroid hormone replacement	No	No	9 months (February–October 2022)

For blood parameters, references ranges are indicated in parentheses and may differ by laboratory. ^a^Values expressed in ng/dl for Patient 1, and in pmol/l for Patient 2 and Patient 3

### Medical history of patient 2

**Figure 2:**
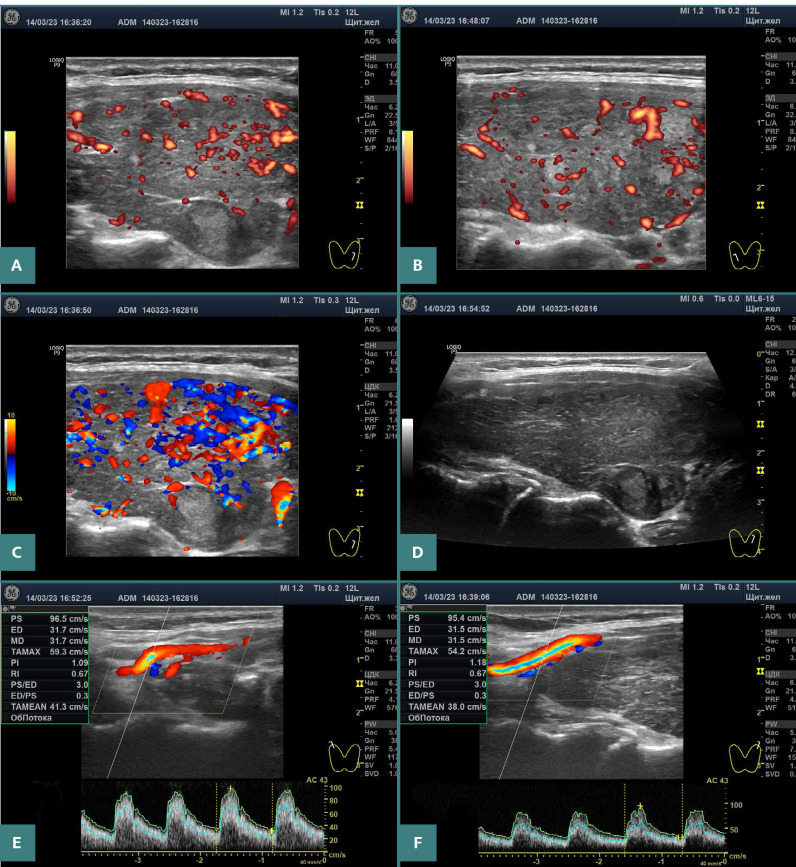
Ultrasound images of the thyroid of patient 2. A,B, Moderate increase in blood flow intensity in two lobes in power Doppler mode at optimal PRF. C, ‘Thyroid inferno’ sign in the left lobe in color Doppler mode at low PRF. D, Left lobe in grayscale mode. E,F, PSV in the first branch of the STAs: 96.5 cm/s on the right and 95.4 cm/s on the left.

In November 2022, the patient became significantly hypothermic during a walk. She said she was “chilled to the bone”, and the wind had a very strong effect on her neck and face. This resulted in frontal neck pain with irradiation to the right ear, and 3 days later, it spread on both sides. Blood tests and additional examinations revealed signs of the first phase of subacute thyroiditis. Within 1 month, the patient received treatment with prednisolone (until 30 December 2022), which led to the restoration of wellbeing, improvement of the ultrasound appearance of the thyroid, and normalization of thyroid hormone metabolism. However, after 2 months (March 2023), the follow-up blood tests revealed manifested hypothyroidism (Table 1). Ultrasonography revealed goiter, increased intensity of thyroid blood flow, and PSV-STA (Figure 2A–F).

### Medical history of patient 3

From 2000 to 2015, the patient experienced substantial mental stress during their tenure in the civil service. In March 2016 and March 2017, she gave birth to two children. In December 2017, GD (hyperthyroidism, TRAb 6.4 IU/l) was diagnosed. For the next few months, she received 50 mg propycil (propylthiouracil). Thyroid hormone metabolism and TRAb levels normalized in the summer of 2018. Between 2018 and 2022, the patient felt fatigued from normal work.

In October 2021, the patient had the following laboratory results: TSH 4.4 mIU/l (normal range, 0.4–4.0), free thyroxine (FT4) 11.3 pmol/l (normal range, 9.00–19.05), free triiodothyronine (FT3) 4.0 pmol/l (normal range, 3.0–5.6), TPOAb >1,000 IU/l (normal range, <5.6), and TGAb 158.4 IU/l (normal range, <4.1).

**Figure 3:**
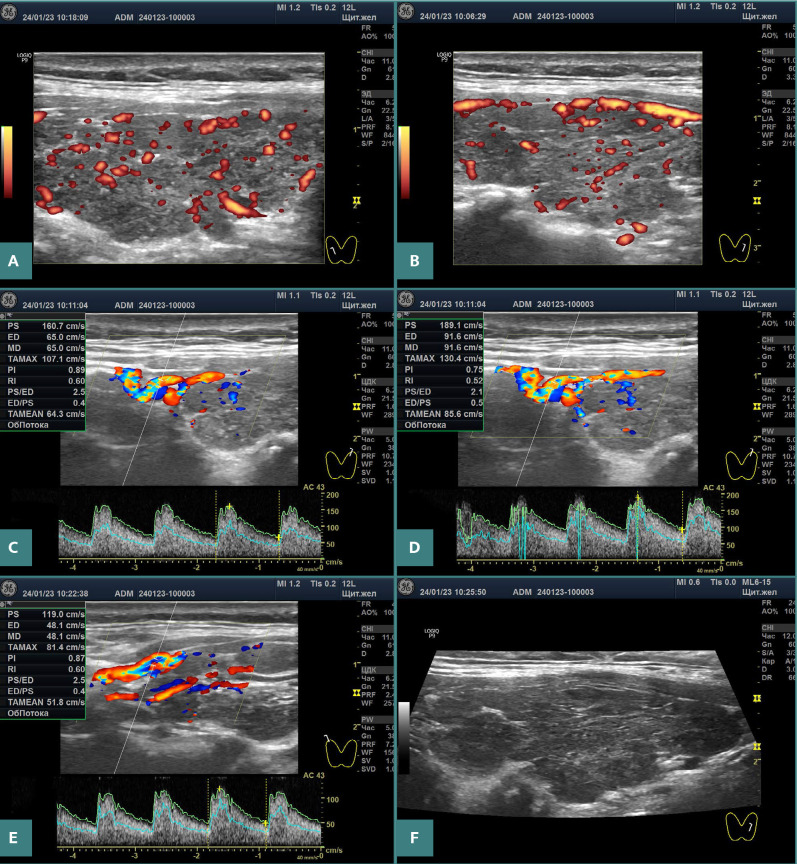
Ultrasound images of the thyroid of patient 3. A,B, Moderate increase in blood flow intensity in two lobes in power Doppler mode at optimal PRF. C, PSV in the first branch of the STAs on the left side at most sites nearly 160 cm/s. D, PSV in the first branch of the STA on the left at one site: 189.1 cm/s. E, PSV in the first branch of the superior thyroid arteries on the right: 119 cm/s. F, Left lobe in grayscale mode.

She had COVID-19 in January 2022 without any fever but had shortness of breath for 1 week. In February 2022, she had the following laboratory results: TSH 50.1 mIU/l (normal range, 0.4–4.0), FT4 <5.4 pmol/l (normal range, 9.00–19.05), FT3 <1.9 pmol/l (normal range, 3.0–5.6), TPOAb >1,000 IU/l (normal values, <5.6), TGAb 158.4 IU/l (normal values, <4.1), and TRAb 1.6 IU/l (normal values, <1.0). She took 75 µg of levothyroxine from February to October 2022, after which she chose not to use hormonal replacement for 3 months before coming to our clinic. Ultrasound examination demonstrated signs of increased activation of thyroid blood flow (Figure 3A–F).

The ultrasound of each of the three patients showed the same pattern: goiter of various grades, marked hypoechogenicity of almost the entire gland (a sign of stromal edema), moderately-to-significantly increased blood flow intensity, and increased PSV of the STA (Figures 1–3 and Table 1).

## DISCUSSION

The conditions of hyperthyroidism and hypothyroidism in GD and primary hypothyroidism identified based on laboratory data are known to convert to one another [15]. Moreover, diffuse hyperthyroidism may develop in patients after many years of primary hypothyroidism [16,17] despite the widespread belief that thyroid becomes underactive in hypothyroidism.

Some studies have reported that hypothyroidism (Hashimoto’s disease) and GD may be the opposite manifestations of the same condition [18]. However, even such a progressive assumption does not elucidate the common pathways of these conditions. This limitation is probably attributed to the following: 1) the ingrained perception of hyperthyroidism (GD) and hypothyroidism as absolute opposites in generations of physicians; 2) the influence of nosological thinking; and 3) adherence to outdated scientific traditions.

Three cases of primary hypothyroidism presented here demonstratively corroborate the pathogenetic link with GD based on ultrasound examinations. The increase in blood flow intensity of the thyroid parenchyma and PSV of the STAs, which are characteristic of GD, was particularly pronounced in these cases (Figures 1–3). In addition, these patients exhibited significant hypoechogenicity and goiter. In other words, the ultrasound pattern in hypothyroidism does not visually differ from that in GD.

Additionally, the ‘thyroid inferno’ phenomenon was observed in the first two patients, with abundant red and blue elements in the color Doppler mode at low pulse repetition frequency (PRF) (Figures 1 and 2). This mode (without PRF optimization) is used by some specialists to perform Doppler examinations. This ultrasound sign is identical to that observed in GD cases.

Previous studies suggested cut-off levels to differentiate primary hypothyroidism from GD based on PSV-STA, with values below 50–70 cm/s and normal values ranging from 26 cm/s to 35 cm/s [13,14]. However, our three hypothyroidism cases had PSV-STA levels of 96–160 cm/s or more (with normal PSV values in the common carotid arteries). Based on these data, an intermediate conclusion can be drawn that most patients with primary hypothyroidism have a relatively small increase in PSV-STA above normal levels. However, in some cases, this increase may be significant. Conversely, in GD, there are more patients with significantly increased PSV-STA values, but there are also cases with only slightly increased PSV-STA values. In other words, the source of thyroid blood flow stimulation in GD often has a more profound effect compared to primary hypothyroidism, but there are no substantial ultrasound differences.

The assumption regarding the effect of TSH or antibodies on thyroid blood flow has not been confirmed. This is also demonstrated by the near absence of TSH in GD and the normal TRAb levels in hypothyroidism. Simultaneously, there is strong evidence of blood flow regulation by the autonomic nervous system [19,20], which may be a key element in the pathogenesis of primary hypothyroidism and GD. From this viewpoint, it can be considered that the intensity of thyroid blood flow and PSV-STA are directly dependent on the magnitude of neurogenic influence on the thyroid parenchyma [21].

In addition, morphological and physiological knowledge of neurothyroid interactions, along with laboratory test data, demonstrate the important role of neurogenic stimulation in thyroid trophism, contributing to goiter and hormone production [20,22,23]. Therefore, it can be inferred that the autonomic nervous system (ANS) regulates the intensity of follicular cell hypertrophy, hormone production, and blood flow. This natural rationality may be justified by the necessity for simultaneous strengthening (or weakening) of thyroid trophism and the transportation of substances in vessels for hormone production.

In primary hypothyroidism, the ANS and TSH induce increased production of thyroid hormones. In GD, the ANS is usually (but not always) accompanied by immune stimulation with Trab instead of TSH. Consequently, the ANS appears to have a key role and is a common pathogenetic basis for both cases of hormone metabolism.

The increased excitation of ANS neurons in our patients was presumably triggered by significant stimuli (mental stress, diseases, hypothermia, etc.) [24–26], as well as by the body’s substantial energy consumption, which required a corresponding increase in calorigenic metabolism, predominantly mediated by thyroid hormones [23]. Therefore, the intense neurogenic stimulation of the thyroid by the ANS was accompanied by ultrasound signs of compensatory parenchymal overload, such as goiter, stromal edema (hypoechogenicity), and increased blood flow intensity and velocity.

Such an ultrasound pattern of the thyroid represents only one aspect of the situation, indicating significant thyroid activation to produce increased amounts of hormones that are intensely consumed by the body under unfavorable conditions. The other aspect is whether this increased hormone production is adequate for the body’s needs. In hypothyroidism, such thyroid stimulation (owing to ANS activation and TSH-induced pituitary activation) only leads to an adequate hormone supply (the quantity of produced T4 and T3 corresponds to the increased need). In GD, the thyroid is overstimulated (owing to ANS activation and TRAb-induced immune system activation) and produces more hormones than the body requires, leading to hyperthyroidism. In other words, hypothyroidism may be deemed a compensatory reaction of the thyroid, and hyperthyroidism can be seen as a hypercompensatory reaction.

By incorporating this important morphofunctional feature (the role of ANS) into our understanding of the disease and recognizing the excessive thyroid stimulation in hypothyroidism, we have, for the first time, managed to integrate disparate data and create a unified nosological–compensatory understanding of these pathological mechanisms. This concept represents the strength of this study. However, the exploration of the neurogenic regulation of the thyroid and of the compensation mechanisms is beyond the scope of this paper. Additional studies will be needed to take into account the identified biases and patterns.

## CONCLUSION

The identical ultrasound manifestations in primary hypothyroidism and diffuse hyperthyroidism (Graves’ disease) indicate a partial coherence in their pathogenesis. This probably involves the leading role of the ANS in thyroid stimulation, which is adequate to meet the body’s hormonal requirements in hypothyroidism and excessive in hyperthyroidism.

## Data Availability

The complete data presented in this study are available on request from the author. The data are not publicly available due to privacy restrictions.
